# The role of catastrophizing in anxiety and depression symptom scores among knee osteoarthritis patients: a multimodal assessment with EEG and machine learning

**DOI:** 10.1007/s00221-026-07368-w

**Published:** 2026-07-31

**Authors:** Jordan Vieira, Marta Imamura, Linamara R. Battistella, Felipe Fregni, Lucas Murrins Marques

**Affiliations:** 1 Mental Health Department, Santa Casa de São Paulo School of Medical Sciences, Dona Veridiana Street 55, 3o. Floor, Higienópolis, São Paulo, SP Brazil; 2 Instituto de Medicina Física e Reabilitação, Hospital das Clínicas HCFMUSP, Faculdade de Medicina, Universidade de São Paulo, São Paulo, SP Brazil; 3 Departamento de Medicina Legal, BioéticaMedicina do Trabalho e Medicina Física e Reabilitação do da Faculdade de Medicina da Universidade de São Paulo (FMUSP), São Paulo, Brazil; 4 Neuromodulation Center and Center for Clinical Research Learning, Spaulding Rehabilitation Hospital and Massachusetts General Hospital, Harvard Medical School, Boston, USA

**Keywords:** Electroencephalography, Knee osteoarthritis, Anxiety, Depression, Pain catastrophizing, Machine learning

## Abstract

To examine dimensional associations between anxiety- and depression-related symptom severity, pain catastrophizing, and resting-state EEG features in patients with knee osteoarthritis (KOA). Resting-state EEG spectral power (delta, theta, alpha, beta) was analysed in 62 KOA patients from the DEFINE cohort. Emotional symptoms were assessed with the Hospital Anxiety and Depression Scale (HADS), along with the Pain Catastrophizing Scale (PCS) and clinical–demographic variables. Multivariate regression analyses identified significant predictors, while linear and tree-based machine learning models were used post hoc to explore whether multivariate and non-linear approaches converged with the regression findings. Catastrophizing was independently and significantly associated with both anxiety and depression symptom scores across regression and exploratory machine learning models. For depression, a multifactorial pattern was additionally observed: higher bilateral parietal delta power, greater catastrophizing, lower education, and greater body weight showed independent associations with more severe symptom scores. Machine learning analyses indicated that EEG features were weak standalone correlates but showed modest complementary associations when combined with clinical variables. Pain catastrophizing was consistently associated with both anxiety and depression symptom scores, and resting-state EEG features showed limited but complementary associations with depressive symptom scores in KOA. Importantly, these associations were observed within a sample presenting predominantly subclinical HADS scores, and findings should be interpreted as reflecting dimensional associations within a rehabilitation cohort rather than clinical anxiety or depressive disorder.

## Introduction

Knee osteoarthritis (KOA) is one of the most common causes of chronic pain and disability worldwide, being a prevalent and disabling chronic condition associated with pain, functional impairment, and reduced quality of life (Hunter and Bierma-Zeinstra 2019). Characterized by progressive degeneration of articular cartilage and associated inflammatory processes, KOA leads to persistent pain, stiffness, and functional decline that severely affects mobility and daily activities. Its global burden is substantial, not only because of its high prevalence in older adults but also due to the increasing number of middle-aged individuals affected, generating long-term socioeconomic impacts (Hunter and Bierma-Zeinstra 2019).

The consequences of knee osteoarthritis extend beyond physical symptoms, encompassing functional limitations, persistent pain, and emotional symptoms such as anxiety and depression within rehabilitation settings. Mental health conditions, particularly depression and anxiety, are highly prevalent among these patients, with studies reporting rates between 20% and 40% depending on disease severity and context (Stubbs et al. 2016). These psychiatric comorbidities interact with pain perception, amplify disability, and reduce adherence to rehabilitation programs (Vargas e Silva et al. 2020), with well-being representing one of the core elements of rehabilitation (Marques and Battistella 2025). Importantly, psychological constructs such as pain catastrophizing—characterized by rumination, magnification of pain-related threat, and feelings of helplessness—play a central role in shaping both the subjective experience of pain and anxiety- and depression-related symptoms in knee osteoarthritis (KOA) and other osteoarthritis populations (Keefe et al. 2001; Sullivan et al. 2001; Somers et al. 2009). Elevated levels of catastrophizing in KOA have been consistently associated with poorer quality of life, greater functional disability, increased pain-related distress, and poorer response to both rehabilitation and pharmacological interventions (Edwards et al. 2011; Riddle et al. 2013).

In recent years, there has been growing interest in identifying neurophysiological markers capable of capturing the interaction between chronic musculoskeletal pain and emotional symptom severity. Electroencephalography (EEG) represents a particularly promising approach due to its non-invasive nature, relatively low cost, and high temporal resolution (Meier et al. 2019). Resting-state EEG oscillatory activity reflects large-scale cortical dynamics related to excitability, sensory integration, and affective regulation, processes that are known to be altered in chronic pain conditions, including knee osteoarthritis.

In osteoarthritis and KOA specifically, alterations in slow-frequency EEG bands such as delta and theta have been linked to disrupted thalamo-cortical communication, impaired inhibitory control, and maladaptive cortical plasticity driven by persistent nociceptive input (Walton et al. 2010; Pinheiro et al. 2016). Conversely, changes in alpha and beta activity have been associated with attentional bias toward pain, hypervigilance, and affective dysregulation in osteoarthritis populations, including individuals with knee osteoarthritis (Jensen et al. 2008; Lim et al. 2016). These oscillatory abnormalities have been shown to correlate not only with pain intensity and duration, but also with anxiety- and depression-related symptoms commonly observed in KOA, supporting the relevance of EEG as a neurophysiological window into pain-related emotional processes.

Identifying reliable EEG markers in KOA represents a critical step toward objective diagnosis of anxiety- and depression-related symptoms. From a clinical perspective, such markers are not intended to replace psychological assessment, but rather to complement self-report measures by capturing neurophysiological variability that may not be fully accessible through subjective reports alone. In rehabilitation settings, this integrative approach may help refine risk stratification and guide future research on personalized interventions.

The present study was designed to investigate associations between resting-state EEG oscillatory activity and emotional symptom severity in patients with knee osteoarthritis. Specifically, we examined whether EEG power across conventional frequency bands (delta, theta, alpha, beta) was associated with anxiety and depression symptoms, while accounting for clinical and demographic factors. Pain catastrophizing was included as a key cognitive–affective explanatory variable, given its established relevance in osteoarthritis-related pain and emotional distress. Our primary analytic strategy relied on multivariate regression models to identify EEG and clinical predictors of anxiety and depression symptom severity. In addition, exploratory machine learning analyses were implemented post hoc to assess whether multivariate and potentially non-linear modeling approaches converged with the regression findings when combining EEG features and clinical variables.

Based on previous literature, we hypothesized that increased low-frequency power (< 13 Hz), especially in frontal regions, would be associated with higher depressive symptoms (Laborde et al. 2011). For anxiety, we expected weaker associations, potentially mediated by catastrophizing rather than direct EEG effects (Higgins et al. 2012). Finally, we anticipated that machine learning models, although exploratory, might reveal multivariate patterns not captured by regression, thereby providing complementary insights into the neurophysiological correlates of anxiety- and depression-related symptoms in KOA.

## Methods and analysis

### Sampling methods, participants, and study design

The present study constitutes a secondary analysis of data from the DEFINE cohort study (Kim et al. 2021), a large-scale research initiative designed to investigate clinical, neurophysiological, and psychosocial aspects of patients undergoing rehabilitation at the Lucy Montoro Rehabilitation Institute (LMRI), part of the Institute of Physical Medicine and Rehabilitation (IPMR) of the Clinical Hospital of the University of São Paulo Medical School, São Paulo, Brazil.

For the current analysis, we focused on the subsample of patients diagnosed with knee osteoarthritis (KOA). Eligible participants were required to be 50 years of age or older, present a clinical and radiological diagnosis of primary KOA according to the Kellgren–Lawrence grading system, and report knee pain (gonalgia) persisting for at least three months. Exclusion criteria included pregnancy, active osteoarthritis with clinical manifestations in regions other than the knee, and the presence of clinical and/or social conditions that could hinder adherence to the rehabilitation protocol. Participants included individuals with unilateral and bilateral knee osteoarthritis, as laterality was not considered a criterion for inclusion.

Between March and December 2019, a total of 113 patients meeting the above criteria were recruited, assessed and systematically collected. All evaluations were performed prior to the COVID-19 pandemic, ensuring that the results presented here are not influenced by pandemic-related restrictions or behavioral changes.

### Demographic and clinical assessment

Demographic and anthropometric information was systematically collected, including age (in years), sex, years of formal education, duration of knee pain (in months), weight (in kilograms), height (in meters), and body mass index (BMI, kg/m^2^). The sex distribution of the sample reflects the epidemiology of knee osteoarthritis, which is known to be more prevalent and clinically severe among women, particularly in older populations. Sex was therefore treated as a potential confounder and systematically tested in all regression models.

Psychological and affective assessments were conducted at baseline, prior to the initiation of the rehabilitation program, ensuring that symptom measures reflected participants’ status at study entry. Participants completed a structured battery of psychological measures administered during a single assessment session.Anxiety and depressive symptoms were assessed using the Hospital Anxiety and Depression Scale (HADS), a 14-item self-report instrument comprising two independent subscales for anxiety and depression, each ranging from 0 to 21 points, with higher scores indicating greater symptom severity. Consistent with established guidelines, scores of 8–10 were interpreted as mild, 11–14 as moderate, and 15–21 as severe anxiety or depressive symptoms. The HADS was the sole instrument used to assess anxiety and depressive symptoms in the present study. Pain catastrophizing was assessed using the Pain Catastrophizing Scale (PCS), a 13-item self-report questionnaire measuring rumination, magnification, and helplessness, with total scores ranging from 0 to 52. All psychological assessments were administered by trained psychologists following standardized protocols, ensuring methodological rigor and reliability.

Finally, considering the potential impact of medications in EEG data, all the medications reported by patients were grouped in the following categories to be considered as independent variables in the models: Antidepressants, Anticoagulant, Anticonvulsants, Benzodiazepines, Neuroleptics, Sleep inducer, Baclofen, Muscle relaxant, Collagen/Phytotherapy, Common analgesic, Opioid, Corticoid, Antihypertensive, Anti Diabetic, Insulin Dependent. Medication classes were included as potential confounders in all regression models and were evaluated following a purposeful selection strategy. Classes that did not significantly contribute to the models or alter the estimated associations between EEG features, catastrophizing, and emotional outcomes were not retained in the final models.

A detailed and complete description of each investigated instrument can be found in Simis et al. (2021).

### Electroencephalography (EEG)

#### Preprocessing

Electroencephalography (EEG) was recorded using a 128-channel EGI Netstation® system, during four minutes of resting state with eyes closed, following the same procedures described in our previous study (Marques et al. 2024). Given the variability in the number of electrodes across systems used in the DEFINE cohort, analyses were restricted to the 21 electrodes common to all recordings, based on the international 10–20 system, in order to facilitate results comparison across studies derived from the DEFINE cohort.

Following the method applied to our previous studies (Marques et al. 2022, 2023), the original raw EEG data were preprocessed following the standardized pipeline PIPEMAT-RS (Preprocessing Integrated Pipeline for Resting-State EEG using MATLAB), which we recently developed and validated to ensure reproducibility and transparency in resting-state analyses (Marques et al. 2025). The pipeline, implemented in MATLAB with EEGLab (Delorme and Makeig 2004) extensions, follows a structured seven-step workflow, based on Makoto’s preprocessing pipeline (Makoto's (2015)). Specifically, our pipeline included the following steps: file format conversion, EEG montage configuration, downsampling and band-pass filtering (1–50 Hz), automated artifact rejection, rereferencing, Independent Component Analysis (ICA), and ICLabel-based component classification for artifact removal.

For the present dataset, preprocessing began with conversion into MATLAB-compatible files and electrode localization according to the 10–20 international system. Data were resampled to 250 Hz, high-pass filtered at 1 Hz, and low-pass filtered at 50 Hz, with additional notch filtering to attenuate powerline noise (60 Hz). The clean_rawdata function was applied to automatically remove flat, noisy, or poorly correlated channels. Datasets were subsequently rereferenced to the average of all electrodes. ICA was performed using the Infomax algorithm. This analysis is effective in identifying artifacts (Delorme et al. 2007; Onton and Makeig 2006) and is significantly related to other techniques, showing greater efficiency in identifying components (Pion-Tonachini et al. 2019). Components associated with ocular, cardiac, muscle, or line noise artifacts were excluded using the ICLabel toolbox (Pion-Tonachini et al. 2019), with a conservative probability threshold of 0.7 for brain activity.

A distinctive feature of PIPEMAT-RS is that each preprocessing step generates a derivative file, allowing complete traceability of the process. This structure improves transparency and facilitates replication by other groups. The full pipeline and validation evidence are available in our prior publication, where we demonstrated its robustness across multiple clinical populations (Marques et al. 2025).

#### Resting-state spectral power analysis

After completing all pre-processing steps, the artifact-free EEG data were analyzed using the pop_spectopo function from EEGLAB, applying a Fast Fourier Transformation (FFT) with 5-s windows and 50% overlap. Relative power, defined as the power within a specific frequency band divided by the total power from 1 to 30 Hz, was calculated for the following bands: Delta (1–3.9 Hz), Theta (4–7.9 Hz), Alpha (8–12.9 Hz), Low Alpha (8–9.9 Hz), High Alpha (10–12.9 Hz), Beta (13–30 Hz), Low Beta (13–19.9 Hz), and High Beta (20–30 Hz). All conventional EEG frequency bands were included to provide a comprehensive characterization of resting-state oscillatory activity. Prior studies examining associations between EEG features and emotional symptoms in chronic pain and osteoarthritis populations have reported heterogeneous and sometimes conflicting findings across frequency bands. Therefore, rather than restricting analyses to a subset of bands a priori, we adopted a systematic screening approach combined with multiple-comparison control to identify the most relevant oscillatory features while limiting spurious associations.

**Fig. 1:**
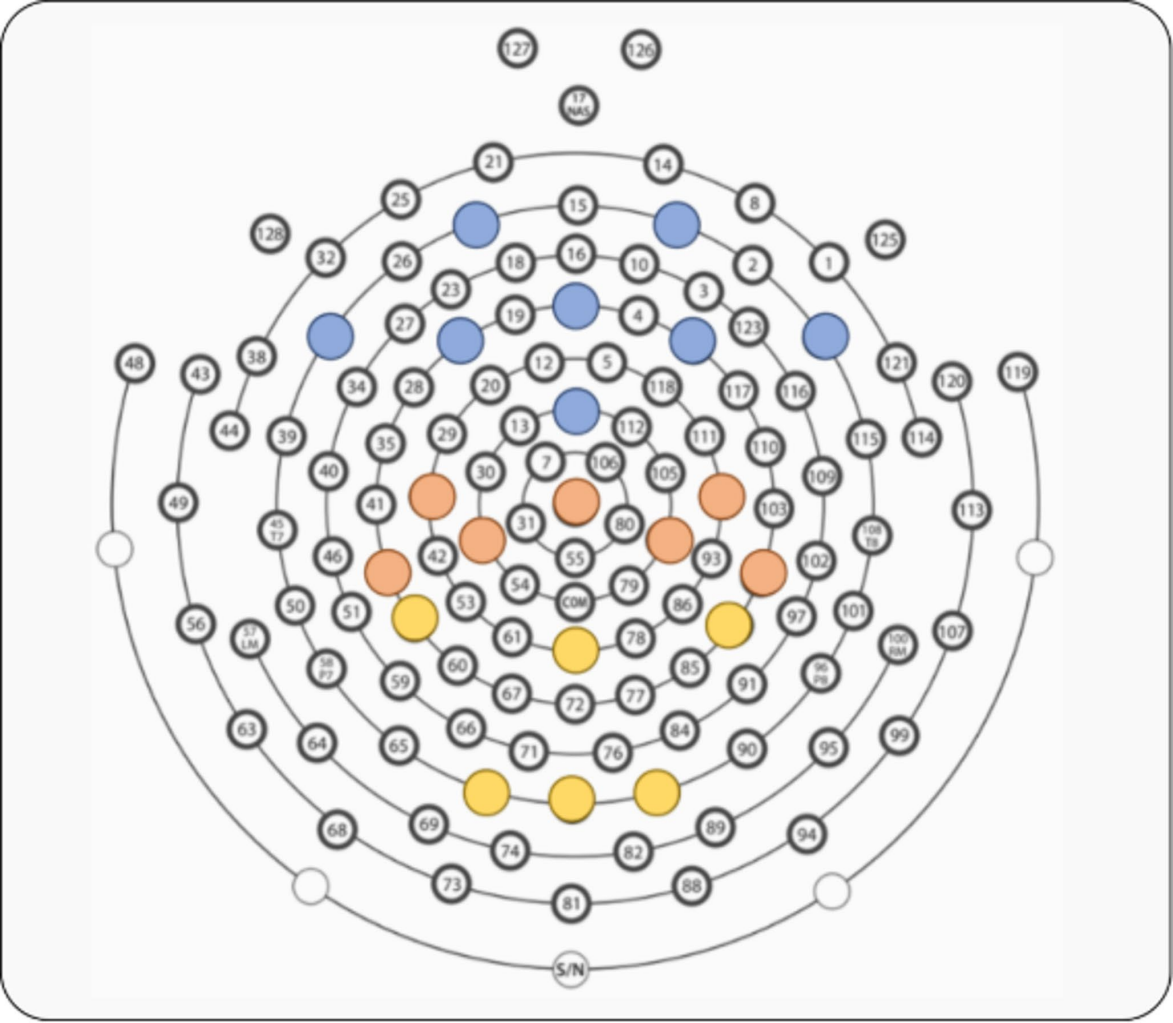
EEG montage considering the 10–20 system. Blue refers to the Frontal region electrodes, orange refers to the Central region electrodes and yellow refers to the Posterior region electrodes

The 21 electrodes (Fig. 1) were then grouped into nine regions of interest (ROIs) based on the international 10–20 system: Frontal Left (E24/F3, E33/F7, and E22/FP1), Frontal Right (E124/F4, E122/F8, and E9/FP2), Frontal Bilateral (E24/F3, E33/F7, E22/FP1, E124/F4, E122/F8, E9/FP2, E6/FCz, and E21/Fz), Central Left (E36/C3, E37/CP1, and E47/CP5), Central Right (E104/C4, E87/CP2, and E98/CP6), Central Bilateral (E36/C3, E37/CP1, E47/CP5, E104/C4, E87/CP2, E98/CP6, and E129/Cz), Posterior Left (E70/O1 and E52/P3), Posterior Right (E83/O2 and E92/P4), and Posterior Bilateral (E70/O1, E52/P3, E83/O2, E92/P4, E75/Oz, and E62/Pz).

Considering the objectives of the present study, which focused on exploring associations between resting-state brain oscillations and emotional symptoms, EEG spectral power was extracted from nine regions of interest (ROIs): frontal left, frontal right, frontal bilateral, central left, central right, central bilateral, posterior left, posterior right, and posterior bilateral. Electrodes corresponding to each ROI were averaged according to the international 10–20 system, as described in the Methods section. For each ROI, relative power was calculated across the frequency bands of interest (Delta, Theta, Alpha, Low Alpha, High Alpha, Beta, Low Beta, and High Beta), resulting in a comprehensive set of 72 EEG variables used in the regression and machine learning analyses. Unlike previous work conducted with stroke patients, in which hemispheric asymmetries were modeled as “affected” and “non-affected,” the present study analyzed bilateral groupings to capture overall spectral dynamics in patients with knee osteoarthritis.

### Statistical analysis

The primary analytic strategy relied on hypothesis-driven multivariate regression models to examine associations between resting-state EEG oscillatory features and emotional symptom severity (anxiety and depression). Pain catastrophizing was consistently modeled as a key explanatory cognitive–affective variable rather than as an outcome.

Given the limited associations observed in regression models, we subsequently conducted exploratory machine learning (ML) analyses as a post hoc complement. The aim of ML analyses was not prediction or confirmation, but to assess whether non-linear or multivariate patterns converged with regression findings. Accordingly, ML results are interpreted cautiously and as exploratory.

#### Primary regression analyses (pre hoc)

All regression analyses were performed using Python (v3.12) and Jamovi® 2.5. Relative EEG spectral power across predefined regions of interest (ROIs) and frequency bands served as independent variables, while anxiety and depression symptom severity (HADS subscales) were modeled as dependent variables in separate analyses.

Demographic and anthropometric variables (age, sex, education, pain duration, weight, height, BMI) were evaluated as potential confounders. Pain catastrophizing was included a priori as a core explanatory variable reflecting cognitive–affective pain processing and was modeled continuously to preserve statistical power. Medication use was evaluated by pharmacological class and retained only if it met predefined confounding criteria.

Univariate linear regressions were first used to screen EEG predictors (p < 0.20). Multivariable models were then constructed using a purposeful selection approach (Bursac et al. 2008), integrating theoretical relevance, confounding assessment (> 10% change in β), and statistical criteria. Backward elimination was applied until only significant predictors and relevant confounders remained. Regression assumptions (linearity, homoscedasticity, independence, normality) were systematically assessed (Osborne and Waters 2002).

Based on prior evidence, we prespecified posterior delta power (depression) and central theta power (anxiety) as primary EEG features of interest. To control for multiple testing during EEG feature screening (9 ROIs × 8 bands), Benjamini–Hochberg FDR correction (q = 0.10) was applied to univariate analyses. Multicollinearity was assessed using variance inflation factors (VIF < 5), influential observations using Cook’s distance (4/n), and heteroskedasticity-robust HC3 standard errors were reported when appropriate. Sensitivity analyses excluded participants using centrally acting medications and re-estimated models adjusted for same-day knee pain (VAS).

Anxiety and depression were modeled separately to avoid multicollinearity and overadjustment, particularly given the modest sample size and conceptual overlap between affective symptom domains.

#### Exploratory machine learning analyses (post hoc)

Machine learning analyses were implemented as an exploratory post hoc complement to the primary regression analyses. All ML analyses were conducted in Python using scikit-learn and mirrored the regression datasets to facilitate direct comparison across analytic frameworks. Outcomes were continuous anxiety or depression scores, consistent with the regression analyses.

For anxiety, three feature sets were evaluated: (i) EEG-only (features passing univariate screening), (ii) PCS-only, and (iii) EEG + PCS. For depression, models compared (i) EEG-only, (ii) confounders-only (PCS, education, and weight), and (iii) EEG + confounders.

Models were evaluated using fivefold cross-validation with shuffled folds (random_state = 42), generating out-of-fold predictions. Performance metrics included R^2^, RMSE, and MAE, with R^2^ summarized as mean values with 95% bootstrap confidence intervals obtained via 1000 bootstrap resamples. Linear models were standardized within folds to prevent data leakage, whereas tree-based models used raw features.

We evaluated ordinary least squares, Ridge, Lasso, ElasticNet, and Random Forest regressors. Hyperparameter tuning for regularized models was nested within cross-validation folds. Model interpretability was explored using SHAP values computed on refitted models. Improvements across feature sets were defined a priori as ΔR^2^ ≥ 0.03 with non-overlapping confidence intervals.

The comparison between regression and machine learning approaches was conceptual rather than inferential, aiming to assess convergence of findings under multivariate and potentially non-linear modeling assumptions rather than formal model comparison or predictive optimization. In line with the exploratory intent and the modest sample size, no external test set or additional complex statistical approaches were implemented to minimize model instability and overfitting. Accordingly, machine learning findings are interpreted as complementary rather than confirmatory.

### Ethical aspects

This study was approved by the local ethics committee. All the participants sign the informed consent form before starting the assessments according to the Declaration of Helsinki (Rickham 1964).

## Results

### Participants

As mentioned in the method section, considering the 113 patients collected, 62 were included as they had the resting-state EEG measure. We tested all patients for outlier profiles (outliers in more than 50% of the dependent variables, above 2.5 standard deviations), and none were excluded from analyses. Table 1 presents the summary of demographic and clinical features of our sample of 62 patients. As expected for a knee osteoarthritis rehabilitation cohort, the sample was predominantly female (91.9%), consistent with epidemiological patterns observed in older adult populations. The characteristics of the eight EEG variables are presented in Table 2.

**Table 1:** Demographic and clinical characteristics

*Demographics*	
Age—mean (SD)	69.23 (± 9.24)
Sex—N (%)	
Male	5 (8.06%)
Female	57 (91.94%)
Weight—mean (SD)	78.18 (± 12.95)
BMI—mean (SD)	31.79 (± 4.77)
Years of education—mean (SD)	12.18 (± 3.70)
*Clinical*	
Time of pain (months)—mean (SD)	100.19 (± 106.86)
HAD (Anxiety)—mean (SD)	5.74 (± 3.91)
HADS (Depression)—mean (SD)	4.66 (± 3.60)
Pain Catastrophizing (PCS)—mean (SD)	14.16 (± 10.95)

Values are presented as mean (± standard deviation) unless otherwise indicated. BMI = Body Mass Index; HADS = Hospital Anxiety and Depression Scale; PCS = Pain Catastrophizing Scale. Time of pain is reported in months

**Table 2:** Description of the power value for each frequency band and hemisphere

Region—oscillation	Bilateral	Left side	Right side
Frontal—Delta	0.15 (± 0.08)	0.15 (± 0.08)	0.15 (± 0.08)
Central—Delta	0.15 (± 0.08)	0.15 (± 0.08)	0.15 (± 0.08)
Posterior—Delta	0.14 (± 0.08)	0.13 (± 0.09)	0.13 (± 0.08)
Frontal—Theta	0.19 (± 0.09)	0.19 (± 0.09)	0.19 (± 0.09)
Central—Theta	0.19 (± 0.08)	0.19 (± 0.08)	0.19 (± 0.09)
Posterior—Theta	0.18 (± 0.09)	0.17 (± 0.09)	0.18 (± 0.09)
Frontal—Alpha	0.42 (± 0.18)	0.42 (± 0.17)	0.41 (± 0.17)
Central—Alpha	0.38 (± 0.15)	0.38 (± 0.15)	0.38 (± 0.15)
Posterior—Alpha	0.44 (± 0.18)	0.43 (± 0.18)	0.44 (± 0.18)
Frontal—LowAlpha	0.23 (± 0.13)	0.23 (± 0.12)	0.22 (± 0.13)
Central—LowAlpha	0.20 (± 0.10)	0.20 (± 0.11)	0.20 (± 0.10)
Posterior—LowAlpha	0.23 (± 0.13)	0.22 (± 0.13)	0.23 (± 0.12)
Frontal—HighAlpha	0.18 (± 0.10)	0.17 (± 0.09)	0.18 (± 0.10)
Central—HighAlpha	0.17 (± 0.08)	0.17 (± 0.08)	0.17 (± 0.08)
Posterior—HighAlpha	0.20 (± 0.11)	0.19 (± 0.12)	0.20 (± 0.11)
Frontal—Beta	0.23 (± 0.10)	0.22 (± 0.10)	0.24 (± 0.10)
Central—Beta	0.27 (± 0.11)	0.27 (± 0.11)	0.27 (± 0.10)
Posterior—Beta	0.23 (± 0.10)	0.25 (± 0.11)	0.23 (± 0.10)
Frontal—LowBeta	0.14 (± 0.06)	0.14 (± 0.05)	0.14 (± 0.06)
Central—LowBeta	0.17 (± 0.06)	0.17 (± 0.07)	0.17 (± 0.06)
Posterior—LowBeta	0.15 (± 0.06)	0.15 (± 0.07)	0.15 (± 0.06)
Frontal—HighBeta	0.09 (± 0.06)	0.09 (± 0.05)	0.09 (± 0.06)
Central—HighBeta	0.10 (± 0.06)	0.09 (± 0.06)	0.10 (± 0.06)
Posterior—HighBeta	0.09 (± 0.06)	0.06 (± 0.06)	0.09 (± 0.06)

Values represent relative spectral power (mean ± standard deviation) for each frequency band and region of interest. Relative power was calculated as the proportion of total power between 1 and 30 Hz. Regions correspond to groupings of electrodes according to the international 10–20 system

### Emotional profile of KOA patients

Following our previously defined objectives and hypotheses, we first investigated the distribution of anxiety and depression symptoms in our sample of patients with knee osteoarthritis (KOA), considering the conventional cutoff score of 8 points on the Hospital Anxiety and Depression Scale (HADS) for clinical relevance. Typically, scores ≥ 8 are adopted as thresholds for identifying clinically relevant symptoms in both subscales of the instrument (anxiety and depression). In our sample of 62 patients, the mean anxiety score was 5.74 (SD = 3.91; range: 0–17), and the mean depression score was 4.66 (SD = 3.60; range: 0–16). We observed that 29.0% of patients scored above the cutoff for anxiety, while 24.2% scored above the cutoff for depression. This distribution indicates that the sample presented a predominantly subclinical symptom profile. This restricted symptom range limits statistical power to detect neurophysiological associations and must be borne in mind when interpreting all subsequent findings.

### Regression analyses

Below we describe the results of the primary multivariate regression analyses examining associations between resting-state EEG spectral power, pain catastrophizing, and anxiety and depression symptom scores in the KOA sample. All regression findings should be interpreted within the context of the predominantly subclinical symptom distribution described above.

#### Anxiety

The initial multivariate regression model for anxiety symptoms included only EEG variables that survived the purposeful selection strategy. This first model (R = 0.418; Adjusted R^2^ = 0.174) retained two significant predictors: increased theta power in the central right region (β = − 127.70, p = 0.004) and increased bilateral central theta power (β = 117.96, p = 0.008).

Given their theoretical and clinical relevance, the previous mentioned variables were subsequently tested as potential confounders. When the Pain Catastrophizing Scale (PCS) was entered into the model, it remained statistically significant (β = 0.131, p = 0.002), leading to the final model. This model (R = 0.549; Adjusted R^2^ = 0.301) explained approximately 30% of the variance in anxiety symptom scores (Table 3). In addition to PCS, both EEG predictors remained significant, although with slightly attenuated coefficients (Central Right Theta: β = − 107.05, p = 0.010; Central Bilateral Theta: β = 100.15, p = 0.016).

**Table 3:** Multivariate model for anxiety levels

Variables	Coefficient	Standard Error	t	p	Adjusted R^2^
Anxiety levels					
Right—Central—Theta	− 107.054	40.184	− 2.66	0.010	0.301
Bilateral—Central—Theta	100.149	40.554	2.47	0.016	
Pain Catastrophizing (PCS)	0.131	0.040	3.25	0.002	

Results from the final multivariate linear regression model predicting anxiety symptoms (HADS-Anxiety)

β coefficients represent unstandardized estimates; β* denotes standardised beta coefficients (computed from unstandardized coefficients and the ratio of standard deviations) to enable valid cross-predictor comparison. Adjusted R2 reflects the proportion of explained variance in the final model

The initial EEG-only model explained 17.4% of the variance in anxiety symptom scores and retained two significant EEG predictors. When pain catastrophizing was subsequently entered, it remained independently significant and improved model fit, while both theta features retained significance with slightly attenuated coefficients. These findings indicate that EEG theta features and pain catastrophizing each contribute independently to anxiety symptom scores.

#### Depression

For depression symptoms, the initial multivariate regression model included EEG variables that met the purposeful selection criteria. The model identified bilateral parietal delta power as a significant predictor (β = 12.69, p = 0.007), suggesting an association between increased slow-wave activity in posterior regions and higher depressive symptoms.

Following this step, all predefined demographic and clinical variables (age, sex, education, weight, height, BMI, and catastrophizing) were systematically tested as potential confounders. In the final model (R = 0.629; Adjusted R^2^ = 0.353), three additional variables were retained as independent predictors: Pain Catastrophizing Scale (PCS; β = 0.097, p = 0.012), education (β = − 0.261, p = 0.022), and weight (β = 0.059, p = 0.046). Together, these factors explained nearly 40% of the variance in depression scores (Table 4).

**Table 4:** Multivariate model for depression levels

Variables	Coefficient	Standard Error	t	p	Adjusted R^2^
*Depression levels*					
Bilateral—Posterior—Delta	12.691	4.495	2.823	0.007	**0.353**
Pain Catastrophizing (PCS)	0.097	0.037	2.589	0.012	
Education	− 0.261	0.111	− 2.345	0.022	
Weight	0.059	0.028	2.041	0.046	

Results from the final multivariate linear regression model predicting depressive symptoms (HADS-Depression)

β coefficients represent unstandardized estimates. Adjusted R2 reflects the proportion of explained variance in the final model

This combined model indicates that parietal delta oscillations are independently associated with depression symptom scores in KOA patients, alongside sociodemographic (education, weight) and cognitive-emotional (catastrophizing) factors. These findings highlight the multifactorial nature of depressive symptom expression in chronic pain, where neurophysiological and clinical characteristics may each contribute complementary associative information.

### Machine learning analyses

Machine learning (ML) analyses were conducted as an exploratory, post hoc complement to the primary regression models. While regression analyses provided interpretable estimates of associations between EEG features, clinical variables, and emotional symptoms, ML approaches were used to examine whether more flexible, multivariate frameworks would converge with these findings under fewer parametric assumptions. Given the modest sample size, the goal of these analyses was not predictive optimization, but rather to probe the robustness and stability of regression results.

All models were implemented in Python using scikit-learn, with fivefold cross-validation to estimate out-of-fold performance. Five algorithms were evaluated: Linear Regression, RidgeCV, LassoCV, ElasticNetCV, and Random Forest Regressor. Three feature configurations were compared: EEG-only, confounders-only, and EEG combined with confounders. Model performance was assessed using R^2^, RMSE, and MAE.

#### Anxiety

For anxiety, ML models were trained using EEG features, pain catastrophizing (PCS), and their combination. Across all algorithms, PCS-only models consistently explained the greatest proportion of variance in anxiety scores, whereas EEG-only models demonstrated near-zero explanatory capacity. Combining EEG features with PCS did not meaningfully improve performance beyond PCS alone.

Importantly, even the Random Forest regressor, designed to capture non-linear effects and interactions, converged on the same pattern, indicating that EEG-derived spectral features provided negligible incremental value once catastrophizing was included. These results closely mirror the regression findings, reinforcing the robustness of catastrophizing as the dominant correlate of anxiety symptoms in this cohort (Table 5).

**Table 5:** Machine learning models for Anxiety levels

Variables	Model	R^2^	RMSE	MAE
*Anxiety levels*				
EEG (only)	ElasticCV	− 0.064	4.003	3.247
EEG (only)	LassoCV	− 0.068	4.011	3.256
EEG (only)	RidgeCV	− 0.291	4.409	3.543
EEG (only)	Random Forest	− 0.125	4.116	3.419
Pain Catastrophizing (PCS) (only)	ElasticCV	0.116	3.649	3.107
Pain Catastrophizing (PCS) (only)	LassoCV	0.109	3.663	3.120
Pain Catastrophizing (PCS) (only)	RidgeCV	0.117	3.646	3.087
Pain Catastrophizing (PCS) (only)	Random Forest	− 0.316	4.453	3.578
EEG + Pain Catastrophizing (PCS)	ElasticCV	0.094	3.694	3.061
EEG + Pain Catastrophizing (PCS)	LassoCV	0.095	3.692	3.067
EEG + Pain Catastrophizing (PCS)	RidgeCV	− 0.203	4.257	3.313
EEG + Pain Catastrophizing (PCS)	Random Forest	0.027	3.828	3.108

Legend: Performance of exploratory machine learning models predicting anxiety symptoms

Models were evaluated using 5-fold cross-validation. EEG-only models include spectral power features only; PCS-only models include Pain Catastrophizing Scale; EEG + PCS models combine both feature sets

R2 = coefficient of determination; ElasticNetCV = ElasticNet with built-in cross-validation; LassoCV = Lasso with built-in cross-validation; RidgeCV = Ridge regression with built-in cross-validation; RMSE = Root Mean Square Error; MAE = Mean Absolute Error

#### Depression

For depression, EEG-only models again showed minimal explanatory capacity across all algorithms. Models based solely on clinical confounders (pain catastrophizing, education, and weight) demonstrated modest but consistently superior performance relative to EEG-only configurations.

The highest stability across folds was observed when EEG features were combined with clinical confounders. Although improvements in R^2^ were small, this pattern suggests that EEG-derived features may provide complementary information when integrated with clinical variables, rather than acting as standalone predictors. This effect was most evident in the Random Forest models, which showed slightly more consistent performance across cross-validation folds (Table 6).

**Table 6:** Machine learning models for depression levels

Variables	Model	R^2^	RMSE	MAE
*Depression levels*				
EEG (only)	ElasticCV	− 0.060	3.680	3.044
EEG (only)	LassoCV	− 0.059	3.678	3.053
EEG (only)	RidgeCV	− 3.573	7.642	5.080
EEG (only)	Random Forest	− 0.091	3.733	3.076
Confounders (only)	ElasticCV	0.173	3.249	2.646
Confounders (only)	LassoCV	0.157	3.281	2.637
Confounders (only)	RidgeCV	0.174	2.249	2.574
Confounders (only)	Random Forest	0.100	3.390	2.714
EEG + Confounders	ElasticCV	0.156	3.283	2.635
EEG + Confounders	LassoCV	0.149	3.298	2.647
EEG + Confounders	RidgeCV	− 2.324	6.515	4.223
EEG + Confounders	Random Forest	0.146	3.303	2.617

Performance of exploratory machine learning models predicting depressive symptoms

R2 = coefficient of determination; RMSE = root mean square error; MAE = mean absolute error

Confounders include Pain Catastrophizing Scale, years of education, and weight. Models were evaluated using 5-fold cross-validation

These machine learning findings should be interpreted with caution. Given the exploratory design, modest sample size (N = 62), and the fact that only a subset of participants exhibited elevated anxiety or depression symptoms, the risk of overfitting remains substantial. Accordingly, ML results are presented as complementary to regression analyses and primarily intended to inform hypothesis generation for future studies in larger, independent samples.

## Discussion

In this study, we investigated the dimensional associations between resting-state EEG oscillations and anxiety and depression symptom scores in patients with knee osteoarthritis (KOA), combining traditional regression modelling with exploratory machine learning approaches. Critically, the sample presented predominantly subclinical HADS scores, with mean anxiety and depression scores well below conventional clinical thresholds; findings must therefore be interpreted as reflecting continuous symptom associations within a KOA rehabilitation cohort rather than clinical disorder. Our results indicated that parietal delta oscillations were independently associated with depression symptom scores, and pain catastrophizing was independently associated with both anxiety and depression symptom scores. Machine learning analyses largely converged with regression findings, indicating that EEG features contributed modest complementary associative information when combined with clinical variables. The present findings should be interpreted within the context of knee osteoarthritis as a chronic pain condition characterised by persistent nociceptive input, functional impairment, and cognitive–affective modulation of pain. The study was designed to explore dimensional associations rather than to discriminate diagnostic groups or identify EEG-based biomarkers of clinical anxiety or depression.

### Anxiety

Our regression analyses revealed that anxiety levels in KOA patients were not primarily explained by EEG oscillatory activity. Although several demographic and clinical variables were systematically tested, only pain catastrophizing (PCS) consistently remained in the final multivariate model. In the present study, pain catastrophizing was conceptualized as a cognitive–affective process that precedes and shapes emotional symptom expression in chronic pain, rather than as an outcome of neurophysiological activity. This framework guided our decision to model catastrophizing as an explanatory variable when examining anxiety and depression.

This finding highlights catastrophizing as a consistently significant and independent correlate of anxiety symptom scores, with EEG power values showing negligible direct associations in univariate screening (Chen & Cros 2009; Ploner et al. 2017). The lack of consistent EEG predictors for anxiety in this cohort may reflect both methodological and clinical factors. Most participants presented anxiety scores below conventional clinical thresholds, limiting symptom variability and statistical power to detect neurophysiological associations. In addition, anxiety in chronic pain contexts may be more strongly explained by cognitive–affective mechanisms—such as pain catastrophizing—than by resting-state oscillatory activity. Finally, anxiety-related neurophysiological signatures may be state-dependent and more likely to emerge during tasks involving threat processing, pain anticipation, or affective reactivity, rather than during eyes-closed resting-state recordings. The association between posterior delta power and depressive symptom severity may reflect altered cortical arousal and inhibitory processes commonly reported in chronic pain conditions. In KOA, sustained nociceptive input and reduced physical activity may contribute to thalamo-cortical dysrhythmia, manifesting as increased slow-wave activity. Such patterns have been linked to depressive symptoms in chronic pain populations and may represent a neurophysiological correlate of pain-related affective burden rather than primary mood disorder pathology.

The exploratory ML models supported this conclusion. Configurations based solely on EEG features displayed poor predictive performance, while models including cognitive-affective variables—particularly PCS—achieved superior accuracy. When EEG variables were added to the confounder set, the models showed minimal improvement, suggesting that EEG may provide complementary but not standalone information (Kim et al. 2017). The consistency between regression and machine learning findings reinforces the importance of catastrophizing as a key correlate of anxiety symptom scores in this KOA cohort, while noting that EEG-only machine learning models showed near-zero or negative cross-validated R^2^ values, indicating poor out-of-fold performance for EEG features in isolation.

From a neurophysiological standpoint, the lack of strong EEG predictors is itself informative. Studies in other clinical populations, such as generalized anxiety disorder, have sometimes reported abnormal frontal alpha asymmetry or altered theta dynamics (Hagger and Chatzisarantis 2011; Bandura et al. 2001). However, in KOA, anxiety levels appear to be better understood through psychological and cognitive mechanisms rather than resting-state oscillatory activity. It is possible that subtle oscillatory changes exist but remain undetected in our sample, given the heterogeneity of patients and the modest sample size (Dyer and Jain 2021; Hunter and Bierma-Zeinstra 2019).

Clinically, these findings reinforce the central role of catastrophizing in the expression of anxiety levels among patients with chronic pain. Targeted psychological interventions, such as cognitive-behavioral approaches aimed at reducing catastrophizing, may be particularly valuable (Peters et al. 2014). Although EEG failed to emerge as an independent biomarker for anxiety, its modest contribution in combined ML models suggests that neurophysiological features could still serve as complementary tools in multimodal assessments, especially in larger studies using more advanced analytical techniques (Schunk et al. 2014; Guedes and Silva 2017).

### Depression

In contrast to the findings for anxiety, our analyses indicated that depression levels in KOA patients showed more consistent associations with neurophysiological features. Specifically, regression models demonstrated that bilateral parietal delta power emerged as a significant predictor of depression levels, even after controlling for demographic and clinical confounders. This result suggests that abnormal slow-wave activity may represent a neurophysiological correlate of depressive symptoms in this chronic pain population (Makeig et al. 2009; Beck and Steer 2013).

The exploratory ML analyses added nuance to this interpretation. Models based exclusively on EEG features performed poorly, similar to the anxiety results, but when EEG data were combined with clinical and cognitive variables, prediction of depression levels modestly improved. Although the incremental gain in explained variance was limited, this pattern suggests that EEG-derived features may provide complementary—rather than primary—information about depressive symptomatology (Beesdo-Baum and Knappe 2018; Cavanagh and Moffitt 2016). Thus, EEG does not stand alone as a robust biomarker but may provide complementary associative information when integrated within multimodal clinical frameworks.

From a neurophysiological perspective, the role of delta oscillations in depression has been documented in other clinical groups, including major depressive disorder, where increased slow-wave activity has been linked to impaired cortical arousal and maladaptive neural inhibition (Aupperle et al. 2015). Alternatively, these delta oscillations may also reflect a compensatory rhythm, representing the brain’s attempt to regulate affective or sensory processing under chronic pain conditions. This dual interpretation highlights the need for further investigation into whether parietal delta activity in KOA patients signals dysfunction, adaptive compensation, or a dynamic balance between the two. Our findings extend this evidence to KOA patients, suggesting that pain-related cortical changes may interact with emotional regulation processes. The specificity of the parietal regions further supports the hypothesis that alterations in sensory integration and attentional networks could underlie depressive manifestations in chronic pain conditions (Li, Hu, Cui, Zhang, & Zhang, 2018). The absence of robust EEG predictors for anxiety may indicate that anxiety symptoms in KOA are more strongly driven by cognitive–affective processes, such as pain catastrophizing, than by resting-state oscillatory activity. Anxiety-related neurophysiological patterns may be more state-dependent and emerge under task conditions involving threat, uncertainty, or pain anticipation, rather than during resting-state recordings.

Clinically, these results highlight the importance of considering both neurophysiological and psychological mechanisms when addressing depression levels in OA. While interventions targeting catastrophizing and affective symptoms remain central, EEG could contribute as a supportive biomarker to stratify risk or monitor treatment effects in research contexts. Importantly, replication in larger samples and with longitudinal designs will be crucial to determine whether resting-state EEG markers such as parietal delta power can truly serve as reliable tools for clinical translation (Guedes and Silva 2017).

### Integrating regression and machine learning

An important aspect of this study was the complementary use of regression and machine learning (ML) approaches. Regression analyses provided clear statistical tests of association, identifying pain catastrophizing (PCS) as a significant and independent correlate of anxiety symptom scores, and parietal delta power as a significant neurophysiological correlate of depression symptom scores. However, regression models, by design, rely on linear assumptions and a stepwise process of variable selection, which may miss subtle or non-linear interactions among features.

In this context, ML analyses were implemented as an exploratory extension to “stress-test” the regression results. For anxiety symptom scores, both regression and ML consistently identified PCS as a significant correlate, while EEG-only machine learning models showed near-zero or negative cross-validated R^2^ values. For depression symptom scores, EEG-only models similarly showed poor cross-validated performance, but when combined with clinical confounders, modest positive R^2^ values emerged for some model configurations, consistent with the regression finding that EEG delta features contribute independently when modelled alongside other clinical variables. The associations between catastrophizing and both anxiety and depression symptom scores were of broadly similar magnitude, and findings should not be interpreted as indicating a categorically stronger role of catastrophizing in anxiety relative to depression.

The integration of these methods also underscores the importance of cautious interpretation. While ML provides a valuable tool for probing multivariate patterns, the relatively modest R^2^ values observed highlight the limitations imposed by sample size and heterogeneity. Overfitting remains a risk, and replication in larger datasets is essential before drawing strong conclusions. Nonetheless, the consistency between regression and ML in pinpointing catastrophizing as central to anxiety and parietal delta oscillations as linked to depression enhances confidence in these findings, while also illustrating how combining statistical and data-driven approaches can strengthen the robustness of exploratory research in clinical neuroscience (Beesdo-Baum and Knappe 2018).

### Clinical implications

Our findings may inform future research and clinical hypothesis development in the context of knee osteoarthritis (KOA). The consistent role of pain catastrophizing in explaining anxiety symptoms reinforces existing evidence that maladaptive cognitive–emotional processes are central to anxiety in KOA, supporting the continued investigation of cognitive-focused approaches within rehabilitation settings. With respect to depressive symptoms, the modest association with parietal delta oscillatory activity suggests that resting-state EEG may provide complementary neurophysiological information when interpreted alongside established clinical and psychological measures. Importantly, these EEG-related findings should not be viewed as standalone indicators, but rather as potential contributors to a broader, integrative understanding of emotional symptom variability in KOA. Given the exploratory nature of the study and the modest sample size, these results do not support immediate clinical application. Instead, they contribute to a mechanistic framework linking cognitive, clinical, and neurophysiological dimensions of emotional symptoms in KOA and may guide the design of future hypothesis-driven studies in larger, independent cohorts.

### Limitations and future directions

This study has several limitations that should be acknowledged. First, the cross-sectional design prevents any causal inference regarding the associations between EEG oscillations and emotional symptoms in KOA patients. Second, although our sample of 62 patients represents a relevant clinical cohort, the modest size limits the generalizability and statistical power of the findings, particularly for exploratory machine learning models. Third, EEG was collected at rest and not during periods of heightened pain or emotional activation, which may have reduced the sensitivity to detect condition-specific oscillatory changes. Moreover, while preprocessing and analysis followed rigorous and standardized procedures (PIPEMAT-RS), more advanced approaches—such as source localization, functional connectivity, or time–frequency dynamics—could provide deeper insights. Also, the predominance of women in the present sample, although consistent with the epidemiology of knee osteoarthritis, limits the generalizability of the findings to male patients. Future studies with more balanced sex distributions will be important to examine potential sex-specific neurophysiological and psychological mechanisms. Although medication use may influence EEG activity and emotional symptoms, this study reflects a real-world clinical cohort in which pharmacological treatment is unavoidable. While medication classes were systematically evaluated as potential confounders and did not materially alter the main findings, residual effects related to dosage, treatment duration, or drug interactions cannot be fully excluded. Future studies with medication-naïve samples or longitudinal designs may further disentangle these effects. We did not perform stratified analyses based on low versus high pain catastrophizing scores due to the limited sample size and the risk of unstable estimates. While effect modification by catastrophizing level is a relevant hypothesis, it will require larger samples specifically powered for subgroup comparisons. The present study did not differentiate between unilateral and bilateral knee osteoarthritis. Although bilateral involvement may be associated with greater pain burden, emotional symptoms and resting-state EEG measures are not joint-specific and were analyzed at the individual level. Future studies may explicitly examine the impact of KOA laterality on neurophysiological and psychological outcomes.

Future research should address these limitations by employing longitudinal designs, larger and more diverse samples, and multimodal approaches integrating EEG with neuroimaging, behavioral, and ecological assessments. Replication with independent datasets and more sophisticated ML frameworks will be critical to validate the robustness and clinical utility of EEG-based markers in KOA.

Finally, future studies with larger samples should formally test mediation and moderation models to examine whether pain catastrophizing mediates or moderates the relationship between neurophysiological markers and emotional symptoms in knee osteoarthritis. Also, a key limitation of this study is the restricted range of anxiety and depression symptoms, with most participants scoring below conventional clinical thresholds. While a subgroup presented clinically relevant symptoms, its size was insufficient to support reliable subgroup or case–control analyses. As a result, findings should be interpreted as reflecting dimensional associations within a KOA rehabilitation cohort rather than diagnostic differentiation. Future studies should include healthy control groups and larger KOA samples stratified by emotional symptom severity to formally test group differences and potential threshold effects.

## Conclusions

In summary, this study provides new evidence on the associations between psychological and neurophysiological factors and emotional symptom scores in patients with knee osteoarthritis, within a sample presenting predominantly subclinical HADS scores. Across analytic strategies, pain catastrophizing was consistently and independently associated with both anxiety and depression symptom scores, underscoring its central role as a modifiable cognitive-emotional correlate. For depression, parietal delta oscillations were identified as a modest but significant and independent correlate, even after controlling for demographic and clinical factors. These slow-wave dynamics may reflect neurophysiological processes related to chronic pain burden rather than primary mood disorder pathology.

Exploratory machine learning models were broadly consistent with regression findings, indicating that catastrophizing was a significant correlate of both outcomes, and that EEG features contributed modest complementary associative value for depression symptom scores when combined with clinical variables.

In conclusion, this secondary and exploratory analysis indicates that pain catastrophizing is a consistently associated cognitive–affective correlate of both anxiety and depression symptom scores in KOA, while resting-state EEG features showed limited but condition-specific associations with depression symptom scores. All findings reflect dimensional associations within a predominantly subclinical sample and should not be generalised to populations with clinically significant anxiety or depressive disorder. Replication in larger and longitudinal cohorts, including samples with greater proportions of clinically relevant symptom scores, will be essential to clarify the neurophysiological mechanisms and to evaluate the potential complementary role of EEG markers in multimodal assessments of chronic pain populations.

## Data Availability

The datasets and code generated and analyzed during the current study are intellectual property of the authors and cannot be made publicly available. However, anonymized datasets and the machine learning model code may be shared with qualified researchers upon reasonable request to the corresponding author.
